# Antiproliferative and cell apoptosis-inducing activities of compounds from *Buddleja davidii* in Mgc-803 cells

**DOI:** 10.1186/1747-1028-7-20

**Published:** 2012-08-31

**Authors:** Jian Wu, Wenshi Yi, Linhong Jin, Deyu Hu, Baoan Song

**Affiliations:** 1 State Key Laboratory Breeding Base of Green Pesticide and Agricultural Bioengineering, Key Laboratory of Green Pesticide and Agricultural Bioengineering, Ministry of Education, Guizhou University, Huaxi District, Guiyang, 550025, China

**Keywords:** *Buddleja davidii*, Anticancer activity, Colchicine, Luteolin

## Abstract

**Background:**

*Buddleja davidii* is widely distributed in the southwestern region of China. We have undertaken a systematic analysis of *B. davidii* as a Chinese traditional medicine with anticancer activity by isolating natural products for their activity against the human gastric cancer cell line Mgc-803 and the human breast cancer cell line Bcap-37.

**Results:**

Ten compounds were extracted and isolated from *B. davidii*, among which colchicine was identified in *B. davidii* for the first time. The inhibitory activities of these compounds were investigated in Mgc-803, Bcap-37 cells *in vitro* by MTT [3-(4,5-dimethylthiazol-2-yl)-2,5-diphenyltetrazolium bromide] assay, and the results showed that luteolin and colchicine had potent inhibitory activities against the growth of Mgc-803 cells. Subsequent fluorescence staining and flow cytometry analysis indicated that these two compounds could induce apoptosis in Mgc-803 cells. The results also showed that the percentages of early apoptotic cells (Annexin V^+^/PI^-^, where PI is propidium iodide) and late apoptotic cells (Annexin V+/PI+) increased in a dose- and time-dependent manner. After 36 h of incubation with luteolin at 20 μM, the percentages of cells were approximately 15.4% in early apoptosis and 43.7% in late apoptosis; after 36 h of incubation with colchicine at 20 μM, the corresponding values were 7.7% and 35.2%, respectively.

**Conclusions:**

Colchicine and luteolin from *B. davidii* have potential applications as adjuvant therapies for treating human carcinoma cells. These compounds could also induce apoptosis in tumor cells.

## Background

*Buddleja* belongs to the Loganiaceae family and has a pantropical distribution across South Asia, Africa, and America [1]. This genus comprises approximately 100 species of wood perennials and shrubs. The roots, leaves, and flowers of various species of *Buddleja* are used in folk medicine in several parts of the world [2]. Various bioactivities, including antimicrobial activity against *Staphylococcus aureus*, as well as antihepatotoxic, antirheumatic, antiprotozoal, and antifungal properties of isolated compounds from *Buddleja* have been reported [3-10]. The application of the poultice or lotion of a number of species of *Buddleja* to treat wounds has also been documented [11,12].

*Buddleja davidii* is a perennial herbaceous plant widely distributed in the Chinese provinces of Yunnan, Guizhou, Sichuan, and Xizang. In Chinese folk medicine, the roots, leaves, and stems of this plant are consumed by drinking an infusion with alcoholic content for the treatment of rheumatism, cough, and fractures.

Studies have evaluated crude extract and different extract partitions from *Buddleja* for their free radical scavenger capacity; neural tissue protection [13]; as well as anticonvulsant [14], antioxidant [15], anti-plasmodium [16], antiviral [17], anti-inflammatory [18,19], and antifungal [9] activities. To the best of our knowledge, the anticancer activity of *B. davidii* has not been studied yet. The aim of this study was to investigate the anticancer property of isolated compounds of *B. davidii*.

The following 10 compounds were isolated from *B. davidii* grown in Guizhou and identified by spectroscopic and physicochemical analysis: luteolin **1**, naringenin **2**, puerarin **3**, rutin **4**, quercetin **5**, hesperetin **6**, and acacetin-7-*O*-α-L-rhamno- pyranosyl(1–6)-β-D-glucopyranoside **7** (flavonoids); stigmasterol **8** (steroid); ferulic acid **9** (phenylpropanoid); and colchicine **10** ( alkaloid ). Colchicine **10** was extracted from *B. davidii* for the first time. All compounds were subjected to bioassay against the human gastric cancer cell line Mgc-803 and the human breast cancer cell line Bcap-37 *in vitro* using the MTT [3-(4,5-dimethylthiazol-2-yl)-2,5-diphenyltetrazolium bromide] method. Luteolin had more potent inhibitory activities against the growth of Mgc-803 cells than the other compounds, and colchicine exhibited high activities against the growth of these cells as well. Further investigations of luteolin and colchicine were thus carried out in Mgc-803 and Bcap-37 cells. Their IC50 values were determined. Fluorescent staining and flow cytometry analysis indicated that both compounds could induce apoptosis in Mgc-803 cells. To the best of our knowledge, this is the first study to report on the apoptosis-inducing and antitumor activities of luteolin and colchicine in Mgc-803 and Bcap-37 cells.

## Results and discussion

### Chemistry

**Figure 1:**
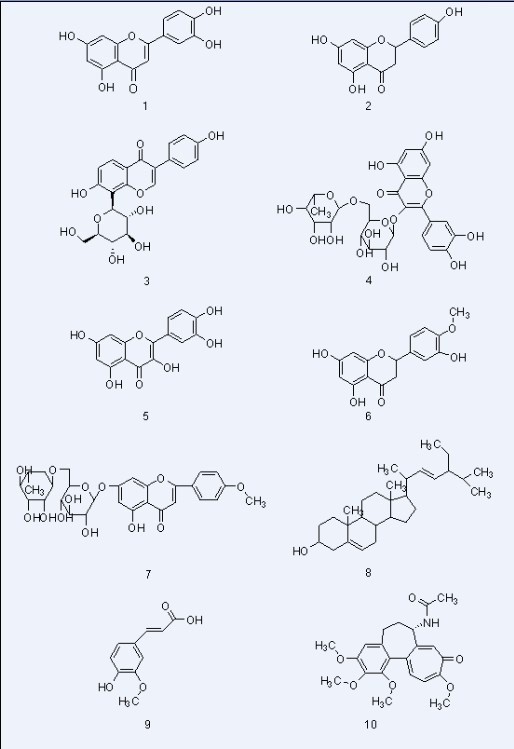
**Structures of compounds 1 to 10.** These compounds were obtained from *B. davidii* and identified by spectroscopic and physicochemical analysis.

Dried *B. davidii* samples collected from Guizhou were studied, and the following compounds were isolated from *n*-butanol extracts, which were identified based on their physicochemical as well as spectroscopic data as luteolin (**1**), naringenin (**2**), puerarin (**3**), rutin (**4**), quercetin (**5**), hesperetin (**6**), acacetin-7-*O*-α-L-rhamno-pyranosyl (1–6)-β-D-glucopyranoside (**7**, flavonoids), stigmasterol (**8**, steroid), ferulic acid (**9**, phenylpropanoid);, and colchicine (**10**, alkaloid). Among them, colchicine (**10**) was obtained from the plants for the first time. All the isolated compounds were shown in Figure 1.

Compound **1** (luteolin [20]), yellow crystal; m.p. 328–330°C; molecular formula: C_15_H_10_O_6_; ESI-MS: *m*/*z* 287 [M + H]+, 309 [M + Na]+; ^1^H NMR (DMSO-d6, 500 MHz) δ: 7.43 (1 H, s, H-2′), 7.40 (1 H, d, *J* = 8.4 Hz, H-6′), 6.89 (1 H, d, *J* = 8.4 Hz, H-5′), 6.68 (1 H, br, s, H-8), 6.45 (1 H, s, H-3), 6.19 (1 H, br, s, H-6); ^13^C NMR (DMSO-d6, 125 MHz) δ: 182.2 (C-4), 164.6 (C-7), 164.4 (C-3), 161.9 (C-9), 157.8 (C-5), 150.2 (C-4’), 146.2 (C-3’), 122.0 (C-1′), 119.5 (C-6’), 116.5 (C-5’), 113.8 (C-2’), 104.2 (C-2), 103.4 (C-10), 98.3 (C-8), 94.3 (C-6).

Compound **2** (naringenin [21]), yellow amorphous crystal; m.p. 253–255°C; molecular formula: C_15_H_12_O_5_; ESI-MS: *m*/*z* 271 [M-H]^-^; ^1^H NMR (CD_3_OD, 500 MHz) δ: 7.28 (2 H, d, *J* = 8.4 Hz, H-2^′^, 6^′^), 6.79 (2 H, d, *J* = 8.2 Hz, H-3′, 5′), 5.86 (1 H, d, *J* = 1.5 Hz, H-8), 5.31 (1 H, d, *J* = 1.4 Hz, H-6), 3.12 (1 H, d, *J* = 0.9 Hz, H-2), 3.06 (1 H, brs, H-3a), 2.65 (1 H, brs, H-3b); ^13^C NMR (CD3OD, 125 MHz) δ: 196.5 (C-4), 167.0 (C-7), 164.1 (C-5), 163.6 (C-9), 157.7 (C-4′), 129.7 (C-1′), 127.7 (C-2^′^, C-6^′^), 114.9 (C-3/), 1 (C-5′), 102.0 (C-10), 95.7 (C-6), 94.8 (C-8), 79.1 (C-2), 42.7 (C-3).

Compound **3** (puerarin [22,23]), white crystal; m.p. 189–191°C; molecular formula: C_21_H_20_O_9_; ESI-MS: *m*/*z* 415 [M-H]^-^, 417 [M + H]^+^, 439 [M + Na]^+^, 455 [M + K]^+^; ^1^H NMR (CD3OD, 500 MHz) δ: 8.03 (^1^H, d, *J* = 9 Hz, H-5), 6.98 (1 H, d, *J* = 9 Hz, H-6), 7.35 (2 H, d, *J* = 9 Hz, H-2, 6), 6.83 (2 H, d, *J* = 9 Hz, H-3, 5), 8.16 (1 H, s, H-2), 5.09 (1 H, d, *J* = 10 Hz, H-1); ^13^C NMR (CD_3_OD, 125 MHz) δ: 176.9 (C-4), 161.7 (C-7, 9), 157.4 (C-4^′^), 153.2 (C-2), 130.1 (C-2^′^, 6^′^), 126.8 (C-1^′^, 5), 124.2 (C-3), 122.9 (C-10), 117.1 (C-3^′^, C-5^′^), 114.9 (C-6), 111.8 (C-8), 81.4 (C-5^′′^), 78.7 (C-3^′′^), 74.3 (C-1^′′^), 71.6 (C-4^′′^), 70.4 (C-2^′′^), 61.4 (C-6^′′^).

Compound **4** (rutin [24,25]), yellow crystal; m.p. 188–190°C; molecular formula: C_27_H_30_O_16_; ESI-MS: *m*/*z* 609 [M-H]^-^, 633 [M + Na]^+^; ^1^H NMR (DMSO-d6, 500 MHz) δ: 7.56 (1 H, d, *J* = 2.0 Hz, H-2^′^), 7.54 (1 H, d, *J* = 2.0 Hz, H-6^′^), 6.86 (1 H, d, *J* = 9.0 Hz, H-5^′^), 6.39 (1 H, d, *J* = 1.9 Hz, H-3^′^), 6.20 (1 H, d, *J* = 1.9 Hz, H-4^′^), 5.36 (1 H, d, *J* = 7.3 Hz, H-1^′′^), 4.54 (1 H, d, *J* = 1.3 Hz, 1^′′′^-H); ^13^C NMR (DMSO-d6, 125 MHz) δ: 177.8 (C-4), 164.6 15 (C-7), 161.7 (C-5), 157.1 (C-9), 156.9 (C-2), 148.9 (C-4^′^), 145.3 (C-3^′^), 133.8 (C-3), 122.1 (C-1^′^), 121.7 (C-6^′^), 116.8 (C-5^′^), 115.8 (C-2^′^), 104.5 (C-10), 101.7 (C-1^′′^), 101.3 (C-1^′′′^), 99.2 (C-6), 94.1 (C-8), 76.3 (C-3^′′^), 76.1 (C-5^′′^), 74.5 (C-2^′′^), 72.3 (C-4^′′′^), 71.0 (C-3^′′′^), 70.9 (C-2^′′′^), 70.5 (C-4^′′^), 68.8 (C-5^′′′^), 67.4 (C-6^′′^), 18.3 (C-6^′′′^).

Compound **5** (quercetin [26]), yellow powder; m.p. 306–308°C; molecular formula: C_15_H_10_O_7_; ESI-MS: *m*/*z* 301 [M-H]^-^, 325 [M + Na]^+^, 341 [M + K]^+^; ^1^ H NMR (DMSO-d_6_, 500 MHz) δ: 7.64 (1 H, d, *J* = 2.3 Hz, H-2^/^), 7.53 (1 H, dd, *J* = 8.6 Hz, *J* = 2.3 Hz, H-6^/^), 6.78 (1 H, d, *J* = 8.6 Hz, H-5^/^), 6.28 (1 H, d, *J* = 2.3 Hz, H-8), 6.20 (1 H, d, *J* = 1.8 Hz, H-6); ^13^C NMR (DMSO-d_6_, 125 MHz) δ: 176.4 (C-4), 164.4 (C-7), 161.2 (C-5), 156.7 (C-9), 148.2 (C-2), 147.3 (C-3^′^), 145.6 (C-4^′^), 136.3 (C-3), 122.5 (C-1^′^), 120.5 (C-6^′^), 116.1 (C-5^′^), 115.6 (C-2^′^), 103.5 (C-10), 98.7 (C-6), 93.9 (C-8).

Compound **6** (hesperetin [27]), white powder; m.p. 216–218°C; molecular formula: C_16_H_14_O_6_; ESI-MS: *m*/*z* 301 [M-H]^-^, 303 [M + H]^+^, 325 [M + Na]^+^; ^1^ H NMR (DMSO-d_6_, 6 500 MHz) δ: 6.91 (1 H, dd, *J* = 8.4, 2.4 Hz, H-6′), 6.90 (1 H, d, *J* = 2.4 Hz, H-2′), 6.88 (1 H, d, *J* = 8.4 Hz, H-5′), 5.87 (1 H, d, *J* = 2.4 Hz, H-8), 5.86 (1 H, d, *J* = 2.4 Hz, H-6), 5.38 (1 H, dd, *J* = 12.1, 2.8 Hz, H-2), 3.73 (s), 3.16 (1 H, dd, *J* = 17.2, 12.1 Hz, H-3b), 2.68 (1 H, dd, *J* = 17.2, 2.8 Hz, H-3a); ^13^C NMR (DMSO-d_6_, 125 MHz) δ: 196.7 (C-4), 167.2 (C-7), 164.0 (C-5), 163.3 (C-9), 148.4 (C-4^′^), 146.9 (C-5^′^), 131.7 (C-1^′^), 118.2 (C-2′), 114.6 (C-6^′^), 112.4 (C-3^′^), 102.3 (C-10), 96.3 (C-6), 95.5 (C-8), 78.8 (C-2), 56.2 (C-7^′^), 42.6 (C-3).

Compound **7** (acacetin-7-*O*-α-L-rhamnopyranosyl(1–6)-β-D-glucopyranoside [28]), 14 yellow powder; m.p. 266–268°C; molecular formula: C_28_H_32_O_14_; ESI-MS: *m*/*z* 593 [M + H]^+^, 615 [M + Na]^+^, 631 [M + K]^+^; 1 H NMR (DMSO-d6, 500 MHz) δ: 12.9 (^1^H, s, 16 OH-5), 8.05 (2 H, dd, *J* = 8.8 Hz, H-3^′^, 5^′^), 7.15 (2 H, dd, *J* = 8.8 Hz, H-2^′^, 6^′^), 6.93 (1 H, S, H-3), 6.79 (1 H, d, *J* = 2.0 Hz, H-8), 6.45 (1 H, d, *J* = 2.0 Hz, H-6), 5.07 (1 H, d, *J* = 7.2 Hz, 18 H-1^′′^), 4.55 (1 H, d, *J* = 1.6 Hz, H-1); ^13^C NMR (DMSO-d_6_, 125 MHz) δ: 182.6 (C-4), 19 164.5 (C-2), 163.5 (C-7), 162.9 (C-5), 161.7 (C-4^′^), 157.1 (C-9), 129.0 (C-2^′^), 128.5 20 (C-6^′^), 123.2 (C-1^′^), 115.3 (C-3^′^), 115.3 (C-5^′^), 105.9 (C-10), 104.4 (C-3), 101.0 (C-1^′′′^), 21 100.4 (C-1^′′^), 100.2 (C-6), 95.3 (C-8), 76.8 (C-3^′′^), 75.7 (C-5^′′^), 73.6 (C-2^′′^), 72.6 22 (C-4^′′′^), 71.3 (C-3^′′′^), 70.9 (C-2^′′′^), 68.4 (C-5^′′′^), 67.7 (C-4^′′^), 66.1 (C-6^′′^), 55.6 (OMe), 1 17.8 (C-6^′′′^).

Compound **8** (stigmasterol [20]), white powder; m.p. 166–168°C; molecular formula: C_29_H_48_O; ESI-MS: *m*/*z* 413 [M + H]^+^; ^1^H NMR (CDCl_3_, 500 MHz) δ: 5.34 (^1^H, br, s, 4 H-6), 5.14 (1 H, dd, *J* = 15.2 Hz, 8.8 Hz, H-22), 5.08 (1 H, dd, *J* = 15.2 Hz, 8.8 Hz, H-23), 3.49–3.53 (1 H, m, H-3), 0.7–2.7 (43 H, m); ^13^C NMR (CDCl_3_, 125 MHz) δ: 140.8 (C-5), 138.4 (C-22), 129.3 (C-23), 121.8 (C-6), 71.9 (C-3), 56.9 (C-14), 56.0 (C-17), 51.3 (C-24), 50.2 (C-9), 42.4 (C-4), 42.3 (C-13), 40.6 (C-20), 39.8 (C-12), 37.3 (C-1), 37.3 (C-10), 32.7 (C-7), 31.9 (C-8), 31.7 (C-25), 31.7 (C-2), 29.0 (C-16), 25.5 (C-28), 24.5 (C-15), 21.3 (C-11), 21.3 (C-21), 21.3 (C-26), 19.5 (C-19), 19.1 (C-27), 12.4 (C-29), 12.1 (C-8).

Compound **9** (ferulic acid [29]), yellow crystal; m.p. 172–174°C; molecular formula: C_10_H_10_O_4_; ESI-MS: *m*/*z* 195 [M + H]^+^, 217 [M + Na]^+^; ^1^ H NMR (DMSO-d6, 500 MHz) δ: 3.89 (3 H, s, OCH3), 6.33 (1 H, d, *J* = 16 Hz, H-8), 6.99 (1 H, d, *J* = 8.4 Hz, H-5), 7.11 (1 H, 14 dd, *J* = 8.4 Hz, H-6), 7.18 (1 H, d, H-2), 7.56 (1 H, d, *J* = 16 Hz, H-7); ^13^C NMR 15 (DMSO-d_6_, 125 MHz) δ: 168.5 (C-1), 149.6 (C-3^′^), 148.4 (C-4/), 145.1 (C-3), 126.3 (C-1^′^), 123.4 (C-6^′^), 116.1 (C-5^′^), 116.0 (C-2), 111.6 (C-2^′^), 56.2 (−OCH_3_).

Compound **10** (colchicine [30]), yellow powder; m.p. 148–150°C; molecular formula: C_22_H_25_NO_6_; ESI-MS: *m*/*z* 400 [M + H]^+^, 422 [M + Na]^+^, 438 [M + K]; ^1^ H NMR (CDCl3, 500 MHz) δ: 7.63 (1 H, s, H-8), 7.35 (1 H, d, *J* = 10.9 Hz, H-12), 6.89 (1 H, d, *J* = 10.9 Hz, H-11), 6.55 (1 H, s, H-4), 4.65 (1 H, dt, H-7), 4.12 (3 H, s, OCH3-10), 3.94 (3 H, s, OCH_3_-2), 3.91 (3 H, s, OCH_3_-3), 3.66 (3 H, s, OCH_3_-1), 2.38–2.01 (1 H, m, H-6);^13^C NMR (CDCl_3_, 125 MHz) δ: 179.6 (C-9), 170.2 (C-13), 164.1 (C-10), 153.6 (C-3), 152.6 (C-7a), 151.3 (C-1), 141.7 (C-2), 137.0 (C-12a), 135.7 (C-12), 134.4 (C-4a), 130.5 (C-8), 125.7 (C-12b), 113.0 (C-11), 107.4 (C-4), 61.7 (OCH_3_-1), 61.5 (OCH_3_ -2), 56.5 (OCH_3_-10), 56.2 (OCH_3_-3), 52.8 (C-7), 36.5 (C-6), 29.9 (C-5), 22.9 (C-14).

### Anticancer activity

The potential effects of the extracts from *B. davidii* on the viability of Mgc-803 and Bcap-37 cells were investigated using MTT assay at 5 and 20 μM, with adriamycin [31] used as the positive control [32,33]. MTT assay is a common method of measuring cell proliferation. The results summarized in Tables 1 and 2 show that luteolin and colchicine possess potent activities against Mgc-803 cells. The activities of luteolin and colchicine at 72 h after treatment were 13.2% ± 4.2% and 26.2%±9.8% against Mgc-803 cells, respectively. And inhibition rates were 50.7%±7.4% and 42.3%±9.6% at 20 μM, respectively. These results indicated that colchicine showed more potent activity against Mgc-803 cells than that of luteolin at 5 μM, but the reverse was true when the concentration was 20 μM. In addition, the results showed that the inhibitory ratios of luteolin against Mgc-803 cells significantly changed compared with colchicine as the concentration increased. Moreover, the synergistic effect of luteolin and colchicine on the cancer cells were also investigated. Unfortunately, the anticancer activity was not obviously found by combination of luteolin and colchicine. For instance, when the concentration was 5 μm, the anticancer activity against Mgc-803 cells were (27.9 ± 8.9)% at 72 h after treatment, whereas the concentration was 20 μm, the inhibitory ratios at 72 h after treatment were (51.3 ± 2.4)% against Mgc-803 cells.

**Table 1:** **Growth inhibitory effects of various constituents of *B. davidii *on different cells at 5** μ**M**

**Compound (5 μM)**	**Growth inhibition (%)**	
	**Mgc-803**	**Bcap-37**
luteolin	13.2 ± 4.2	9.6 ± 6.8
naringenin	2.3 ± 2.9	5.3 ± 5.4
puerarin	0.4 ± 5.4	5.3 ± 7.6
rutin	8.3 ± 7.1	0.4 ± 3.7
quercetin	29.2 ± 4.1*	12.1 ± 8.2
hesperetin	0.0 ± 2.4	7.9 ± 6.4
acacetin-7-O-α-L- rhamnopyranosy (1–6)- β-D-glucopyranoside	0.5 ± 4.3	0.9 ± 5.2
stigmasterol	0.8 ± 3.1	1.3 ± 7.4
ferulic acid	0.9 ± 6.0	8.2 ± 5.8
colchicine	26.2 ± 9.8*	19.4 ± 5.3*
Adriamycin	62.5 ± 4.6**	47.3 ± 5.5**

*P<0.05 and **P<0.01 versus control.

**Table 2:** **Growth inhibitory effects of various constituents of *B. davidii *on different cells at 20** μ**M**

**Compound (20 μM)**	**Growth inhibition (%)**	
	**Mgc-803**	**Bcap-37**
Luteolin	50.7 ± 7.4**	28.8 ± 3.0*
Naringenin	4.9 ± 3.0	12.0 ± 3.2
Puerarin	3.5 ± 7.0	11.1 ± 5.3
Rutin	21.0 ± 10.3*	7.3 ± 4.9
Quercetin	31.2 ± 6.2*	16.1 ± 6.8
Hesperetin	2.9 ± 4.8	10.6 ± 3.1
acacetin-7-O-α-L-rhamnopyr-anosy (1–6)- β-D-glucopyranoside	4.2 ± 4.6	10.0 ± 4.0
stigmasterol	4.7 ± 5.1	2.6 ± 8.7
ferulic acid	3.9 ± 5.2	30.2 ± 5.8*
colchicine	42.3 ± 9.6**	26.5 ± 6.2*
Adriamycin	92.8 ± 1.0**	89.9 ± 1.3**

*P<0.05 and **P<0.01 versus control.

**Figure 2:**
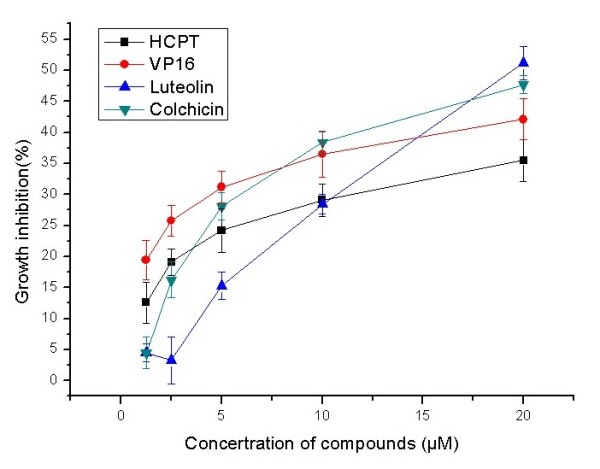
**Effects of compounds on proliferation of Mgc-803 cells.** After Mgc-803 cells were treated with HCPT, VP-16, luteolin, and colchicine for 72 h in varying concentrations (1.25 to 20 μM), their growth inhibition was detected using MTT assay. Data are presented as mean±SD, *n* = 3.

**Figure 3:**
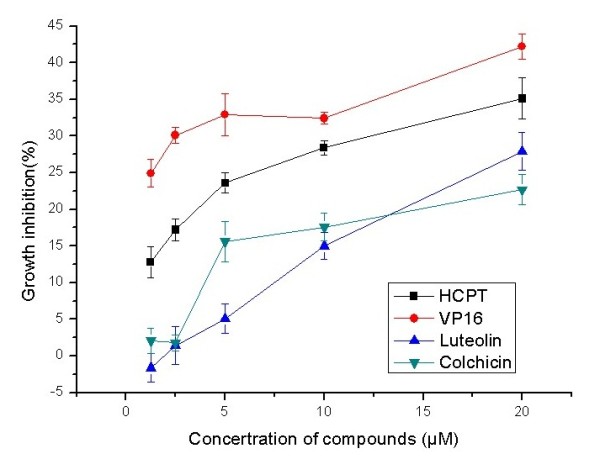
**Effects of compounds on proliferation of Bcap-37 cells.** After Bcap-37 cells were treated with HCPT, VP-16, luteolin, and colchicine for 72 h in varying concentrations (1.25 to 20 μM), their growth inhibition was detected using MTT assay. Data are presented as mean±SD, *n* = 3.

Further experiments also found that proliferation of Mgc-803 cells was significantly 4 inhibited by luteolin and colchicine, as shown in Figures 2 and 3. With hydroxycamptothecine (HCPT) and etoposide (VP-16) as positive controls, the IC_50_ values of luteolin and colchicine on Mgc-803 cells were 19.87±1.0 and 18.79±1.6 μM, respectively, and IC_50_ values of luteolin and colchicine on Bcap-37 cells were 41.78±2.2 and 76.01±0.6 μM, respectively. The results also showed that luteolin and colchicine had more potent activities against Mgc-803 cells. Besides, the inhibitory effect on Bcap-37 cells of luteolin was stronger than that of colchicine.

Apoptosis is a physiological pattern of cell death characterized by morphological features and extensive DNA fragmentation, the frequency and time of appearance of which depend on the cell line and the apoptosis-inducing signal. It has been well studied that luteolin is capable of inducing cell cycle arrest or apoptosis in various human cancer cells [34-40], such as HT-29 human colon cancer [34], hepatoma cells [35,36] , human myeloid leukaemia cells [37], human lung squamous carcinoma CH27 cell [38], and so on. Moreover, Colchicine can induce cytoskeletal collapse and apoptosis in N-18 neuroblastoma [41] and showed Anti-Mitotic Activity [42]. In order to preliminarily determine the action of luteolin and colchicine, changes in the morphological character of Mgc-803 cells were investigated using acridine orange (AO)/ethidium bromide (EB) staining, Hoechst 33258 staining, and TUNEL (terminal deoxynucleotidyl transferase biotin-dUTP nick end labeling) staining under fluorescence microscopy to determine 3 whether the growth inhibitory activities of luteolin and colchicine were related to the 4 induction of apoptosis.

**Figure 4:**
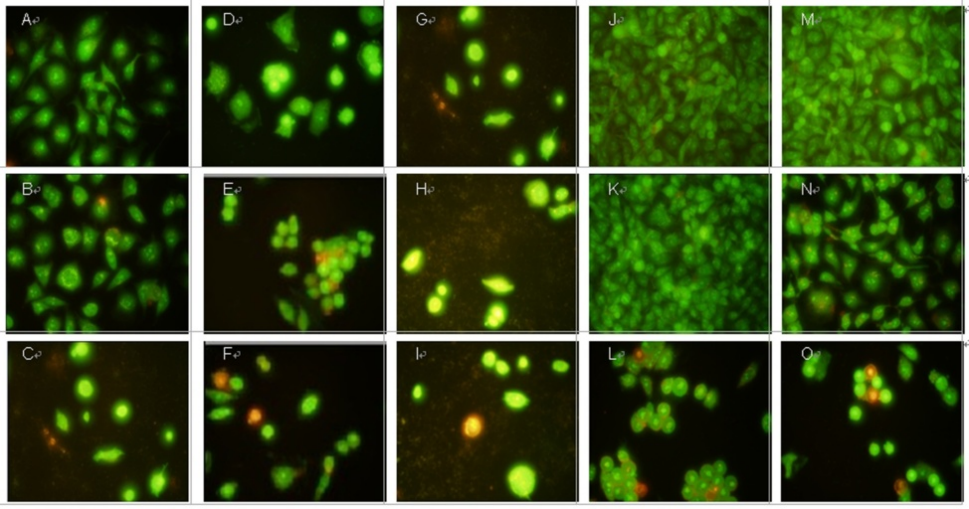
**Morphological changes in the nuclei during luteolin- and colchicine-induced apoptosis in Mgc-803 cells detected by AO/EB staining.** After treatment with colchicine and luteolin at 5, 10, and 20 μM, Mgc-803 cells were stained with AO/EB (100 μg/μM) and observed under fluorescence microscopy. **A**: Negative control (without treatment). **B, C**: Positive control; treatment with VP-16 and HCPT (20 μM) for 24 h, respectively. **D–F**: Treatment with colchicine (5, 10, and 11 μM, respectively) for 24 h. **G–I**: Treatment with colchicine (5, 10, and μM, 12 respectively) for 36 h. **J–L**: Treatment with luteolin (5, 10, and 20 μM, respectively) for 24 h. **M–O**: Treatment with luteolin (5, 10, and 20 μM, respectively) for 48 h.

Since AO is a crucial dye and can stain nuclear DNA across an unbroken cell membrane, whereas EB can only stain cells that had lost an intact cell membrane [43]. Thus, the following phenomena were observed after AO/EB staining: (1) viable cells have been uniformly stained green; (2) early apoptotic cells have been stained green 9 yellow or displayed green yellow fragments; (3) late apoptotic cells have been stained orange or displayed orange fragments; and (4) necrotic cells have been stained orange to red fluorescing nuclei with no indication of chromatin fragmentation. As shown in Figure 4, all the morphological changes were observed after Mgc-803 cells were treated with luteolin and colchicine for 24, 36, and 48 h. Green live Mgc-803 cells with a normal morphology were seen in the negative control group (Figure 4A). In contrast, early apoptotic cells with yellow green dots and late apoptotic cells with orange dots in Mgc-803 cell nuclei could be seen in the positive control group (Figure 4B and C). At the same time, bright green early apoptotic cells with nuclear margination and chromatin coagulation were seen in the experimental group (Figure 4D, G, J, K, M, and 19 N), as were orange late apoptotic cells with apoptotic bodies and chromatin fragmentation (Figure 4E, F, H, I, L, and O). The results suggested that both of colchicine and luteolin were able to induce apoptosis in Bcap-37 cells. Moreover, it can be seen that colchicine showed more apoptotic activity against Mgc-803 cells than that of luteolin.

**Figure 5:**
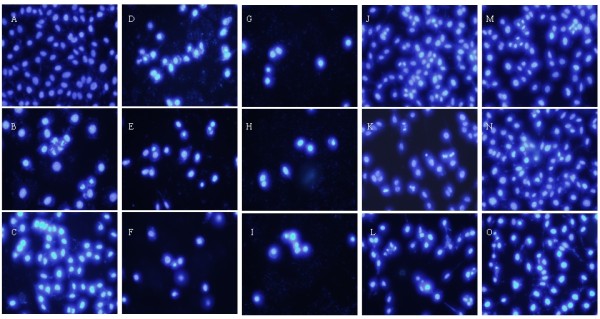
**Morphological changes in the nuclei during luteolin- and colchicine-induced apoptosis in Mgc-803 cells detected by Hoechst 33258 staining.** After treatment with colchicine and luteolin at 5, 10, and 20 μM, Mgc-803 cells were stained with Hoechst 33258 and observed under fluorescence microscopy. **A**: Negative control (without treatment). **B, C**: Positive control; treatment with VP-16 and HCPT (20 μM) for 48 h. **D–F**: Treatment with colchicine (5, 10, and 20 μM, respectively) for 24 h. **G–I**: Treatment with colchicine (5, 10, and 20 μM, respectively) for 36 h. **J–L**: Treatment with luteolin (5, 10, and 20 μM, respectively) for 24 h. **M–O**: Treatment with luteolin (5, 10, and 20 μM, respectively) for 48 h.

Membrane-permeable Hoechst 33258 (a blue fluorescent dye with low cytotoxicity) staining was also performed to investigate the apoptosis-inducing activities of colchicine and luteolin in Mgc-803 cells. Live cells with homogeneous light blue nuclei could be observed under fluorescence microscopy after staining with Hoechst 33258, and the apoptotic cells displayed bright blue due to karyopyknosis and chromatin condensation after treating with Hoechst 33258, and the nuclei of dead cells could not be stained [43]. As shown in Figure 5, all the mentioned changes could be observed. Compared with the negative control (Figure 5A), a proportion of cells with smaller nuclei and condensed staining appeared in the positive control group (Figure 5B and C). Some Mgc-803 cells nuclei underwent pyknosis after treatment with colchicine for 24 and 36 h, as shown in Figure 5D–I, as did some Mgc-803 cell nuclei after being treated with luteolin for 24 and 36 h, as shown in Figure 5J–O. These results confirmed that colchicine and luteolin could induce apoptosis in Mgc-803 cells and that colchicine had more potent activity against Mgc-803 cells than luteolin.

**Figure 6:**
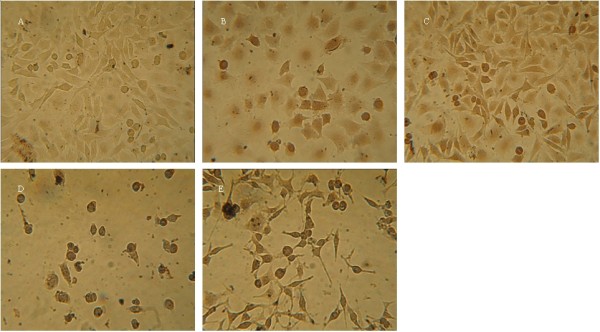
**Morphological changes during luteolin and colchicine-induced apoptosis in Mgc-803 cells detected by TUNEL staining.** After treatment with colchicine and luteolin at 20 μM, Mgc-803 cells were stained with TUNEL and observed under light microscopy. **A**: Negative control (without treatment). **B, C**: Positive control; treatment with VP-16 and HCPT (20 μM) for 24 h. **D**: Treatment with colchicine (20 μM) for 24 h. **E**: Treatment with luteolin (20 μM) for 24 h.

TUNEL staining was further carried out to confirm the cell apoptosis-inducing activities of colchicine and luteolin. TUNEL staining is a common method for detecting DNA fragmentation that results from apoptotic signaling cascades [44]. Apoptotic cells thus showing a brown color [43]. The experimental results are demonstrated in Figure 6.

From the figure, it could be found that most nuclei in the treatment groups with VP-16 (Figure 6B), HCPT (Figure 6C), colchicine (Figure 6D), and luteolin (Figure 6E) were stained as a discernible brown compared with the control (Figure 6A).

**Figure 7:**
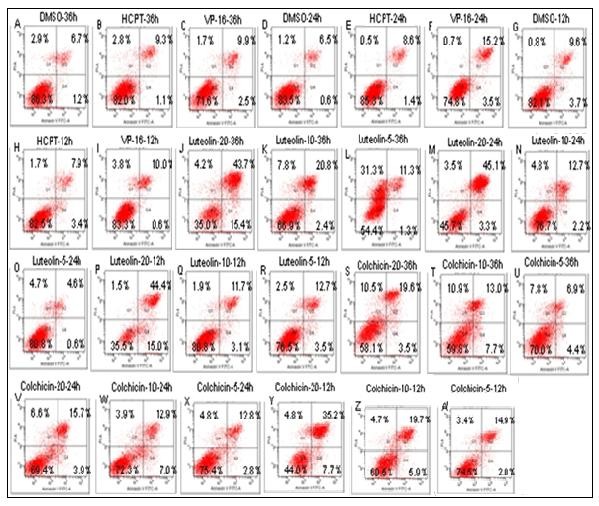
**Flow cytometry analysis for apoptosis-inducing activities of colchicine and luteolin in Mgc-803 cells.** The appearance of apoptosis cells was detected by flow cytometry using Annexin V/PI staining. **A–C:** Negative control. **D–I:** Positive control; treatment with HCPT and VP-16 (20 μM) for 12, 24, and 36 h. **J–R:** Treatment with luteolin (5, 10, and 20 μM) for 12, 24, and 36 h. **S–A:** Treatment with colchicine (5, 10, and 20 μM) for 12, 24, and 36 h.

Moreover, the apoptosis ratios induced by colchicine and luteolin caused apoptosis in the Mgc-803 cells were analyzed by flow cytometry with Annexin V/propidium iodide (PI) double staining after treating with colchicine and luteolin for 12, 24, and 36 h. Exposure of the membrane phospholipid phosphatidylserine to the external cellular environment is one of the earliest markers of apoptotic cell death. Annexin V is a calcium-dependent phospholipid-binding protein with high affinity for phosphatidylserine expressed on the cell surface used to differentiate intact cells (Q3; Annexin V^-^/PI^-^). PI does not penetrate whole cells with intact membranes and was used to differentiate between necrotic cells (Q1; Annexin V^-^/PI^+^), late apoptotic cells (Q2; Annexin V^+^/PI^+^), and early apoptotic cells (Q4; Annexin V^+^/PI^-^). The results are shown in Figure 7.

As indicated in Figure 7, the percentages of Q4 (Annexin V+/PI^-^) and Q2 (Annexin V^+^/PI^+^) were approximately 0.6% and 4.6% at 5 μM, 3.1% and 11.7% at 10 μM, and 15.0% and 44.4% at 20 μM, respectively after 12 h treating with luteolin. While the percentages that were treated for 24 h were approximately 3.5% and 12.7% at 5 μM, 2.2% and 12.7% at 10 μM, and 3.3% and 45.1% at 20 μM for Q4 and Q2 cells, respectively. In addition, the corresponding values of treating for 36 h were approximately 2.4% and 20.8% at 5 μM, 1.3% and 11.3% at 10 μM, and 15.4% and 43.7% at 20 μM, respectively. When the experiments were treated with colchicine, the percentages (after treating 12 h) of Q4 and Q2 cells were approximately 4.4% and 6.9% at 5 μM, 7.0% and 12.9% at 10 μM, and 3.9% and 15.7% at 20 μM, respectively. When the time was extended to 24 h, the corresponding percentages were approximately 2.8% and 12.8% at 5 μM, 7.7% and 13.0% at 10 μM, and 3.5% and 19.6% at 20 μM, respectively. Moreover, the corresponding percentages (after treating for 36 h) were approximately 2.0% and 14.9% at 5 μM, 5.9% and 19.7% at 10 μM, and 7.7% and 35.2% at 20 μM, respectively. These results showed that luteolin and colchicine may exert their anticancer activities in Mgc-803 cells by interference of cell proliferation via apoptosis in a dose- and time-dependent manner.

In summary, the inhibitory effects observed in response to colchicine and luteolin were associated with the induction of apoptotic cell death.

## Conclusions

*B. davidii*, a class of Chinese traditional medicine, which showed variety of biologically activity and is widely distributed in southwestern of China. Studies on the chemical constituents of *B. davidii* and their biological activities have focused on the rational development and utilization of this plant.

In the current study, 10 compounds were extracted and identified from *B. davidii* grown in Guizhou, and their cell growth inhibitory effects on Mgc-803 and Bcap-37 cells were evaluated by MTT assay. Among these ten compounds, colchicine **10** was extracted from *B. davidii* firstly. Both of colchicines and luteolin showed potent anticancer activities on Bcap-37 and BGC-823 cells in a dose-dependent manner. And the IC50 values of luteolin and colchicine on Mgc-803 cells were 19.87±1.0 and 18.79±1.6 μM, respectively, The IC50 values of luteolin and colchicine on Bcap-37 cells were 41.78±2.2 and 76.01±0.6 μM, respectively. This is the first study to have extracted colchicine from *B. davidii*. Moreover, the apoptotic activities induced by colchicine and luteolin in Mgc-803 cells were investigated through AO/EB staining, Hoechst 33258 staining, TUNEL assay, and flow cytometry analysis. The results demonstrated that both compounds are promising adjuvant therapies for treating human carcinoma cells. Further studies are needed to clarify the action mechanism of *B. davidii* on the inhibition of human malignant tumor cell proliferation.

## Methods

### Plant materials

Fresh *B. davidii* samples were collected from Bijie, Guizhou, in 2008. The plant was identified by Professor Deqing Long (Guiyang Medical University). A voucher specimen was deposited in the Botany and Pharmacognosy Department, School of Pharmacy, Guiyang Medical University.

### Extraction and isolation

Dried *B. davidii* (20 kg) samples were powdered and extracted with ethanol at room temperature four times for 7 days each. After filtration, the extract was evaporated under reduced pressure for ethanol removal to obtain a water suspension that was sequentially extracted at room temperature with petroleum ether (5000 mL), ethyl acetate (5000 mL), *n*-butanol (5000 mL), and water (5000 mL).

The *n*-butanol extract of *B. davidii* (195 g) was chromatographed on a Si gel column (1800 g, 200–300 mesh) eluted with ethyl acetate/methanol mixtures. The fractions eluted with a 10:1 ethyl acetate/methanol ratio yielded 10.2 g of extract A, whereas those eluted with a 5:1 ethyl acetate/methanol ratio afforded 15.4 g of extract B. Extract A was subjected to passage over a Si gel column (250 mL) and eluted with trichloromethane, trichloromethane/methanol, and methanol. It was fractionated into three parts (A1–A3): The A1 fraction was purified on Merck Silica gel 60RP-18 (30%,40%, and 50% methanol) to obtain compound **10** (18 mg) and compound **8** (60 mg). The A2 fraction was purified on a Sephadex LH-20 column (methanol/water) and then a Si gel column (trichloromethane/methanol, 90:7) to obtain compound **9** (43 mg). The A3 fraction was purified on Si gel column (trichloromethane/methanol, 70:3) to obtain compound **2** (30 mg).

Extract B was fractionated into four parts (B1–B4) over a Si gel column (350 mL) eluting with trichloromethane/methanol (30:1): The B1 fraction was purified on Merck Silica gel 60RP-18 (30%, 40%, 50%, and 100% methanol) to obtain compound **6** (25 17 mg) and compound **5** (63 mg). The B2 fraction was purified on Merck Silica gel 18 60RP-18 (40%, 50%, and 100% methanol) to obtain compound **3** (31 mg) and compound **1** (24 mg). The B3 fraction was purified on a Si gel column (trichloromethane/methanol, 10:7) to obtain compound **7** (160 mg). The B4 fraction was purified on a Sephadex LH-20 column (methanol) to obtain compound **4** (42 mg).

### Cell lines and culture

The human gastric cancer cell line Mgc-803, human breast cancer cell line Bcap-37 were grown in RPMI (Roswell Park Memorial Institute) 1640 and supplemented with 10% fetal bovine serum at 37°C in a humidified atmosphere with 5% CO_2_; All cell lines were purchased from Institute of Biochemistry and Cell Biology, Shanghai Institute for Biological Sciences, Chinese Academy of Sciences.

### Anticancer activity assays

#### MTT assay

The activities of the compounds in Mgc-803, Bcap-37 were evaluated *in vitro* using MTT assay. All cell lines (2000 cells/well) were incubated for 24 h at 37°C in an atmosphere of 5% CO_2_ before the compounds were added. After treatment with various concentrations of each extract, the cells were incubated for an additional 72 h at 37°C in 5% CO2. Subsequently, the medium was removed and the cells in each well were incubated with 100 μL of MTT solution (0.5 mg/mL) for 4 h at 37°C. Finally, the mixture was supplemented with sodium dodecyl sulfate and the cells were incubated for 12 h at 37°C.

All experiments were performed in triplicate. The inhibition rate was calculated as follows:

(1) $$Growth\;inhibitionrate\;\left(\%\right)=\left(\frac{A_{\mathrm{control}}-A_{\mathrm{drug}}}{A_{\mathrm{control}}}\right)\times 100$$

#### AO/EB double staining

Apoptotic morphology was investigated by double staining with AO and EB as described by Baskic et al. [45]. In the experiment, Mgc-803 cells were seeded in six-well plates at 5 × 104 cells per well in 0.8 mL of RPMI 1640 medium supplemented with 10% fetal bovine serum and cultured for 24 h, followed by removal of the culture medium, replacement with fresh medium plus 10% fetal bovine serum, and supplementation with puerarin and colchicine. After the treatment period, the cover slips with monolayer cells were inverted on a glass slide with 20 μL of AO/EB stain (100 μg/mL). Fluorescence was read on an IX71SIF-3 fluorescence microscope (OLYMPUS Co., Japan).

#### Hoechst 33258 staining

Mgc-803 cells grown on sterile cover slips in six-well tissue culture plates were treated with puerarin and colchicine for a certain range of time. The culture medium containing compounds was removed, and the cells were fixed with 4% paraformaldehyde for 10 min. After washing twice with PBS, the cells were stained with 5 μg/mL of Hoechst 33258 for 5 min. After rewashing twice with PBS, the percentage of apoptotic cells was determined using an IX71SIF-3 fluorescence microscope. As some of the dead cells were rinsed off as the experiment progressed, the apoptotic ratios could have been underestimated in comparison with those from the MTT assay.

#### TUNEL staining

TUNEL assay identifies apoptosis by tagging the 3’-OH ends of DNA fragments with fluorescein [41]. In the present study, Mgc-803 cells grown in six-well tissue culture plates were treated with puerarin and colchicine. The cells were subsequently washed once in 1 mL of PBS and fixed in 4% paraformaldehyde for 60 min. After another round of washing with PBS, the cells were incubated with Immunol staining wash buffer (Beyotime) on ice for 2 min. Cells were rewashed once with PBS and then incubated in 0.3% H_2_O_2_ in methanol at room temperature for 20 min to inactivate the endogenous peroxidases, after which the cells were washed three times with PBS. Thereafter, the cells were incubated with 2 μL of terminal deoxynucleotidyl transferase enzyme and 48 μL of biotin-dUTP per specimen for 60 min at 37°C. After termination for 10 min, the cells were incubated with 50 μL of streptavidin-HRP for 30 min at room temperature after being washed thrice with PBS.

#### Flow cytometry analysis

For measuring apoptosis, Mgc-803 cells were seeded in six-well plates at a density of 5 × 10^5^ cells/mL for 24 h and then treated with puerarin and colchicine at 5, 10, and 20 μM. After 12, 24, or 36 h, the cells were collected, washed twice with PBS, and centrifuged at room temperature. Subsequently, the Mgc-803 cells were gently resuspended in 500 μL of binding buffer. Thereafter, the cells were stained in 5 μL of Annexin V/FITC and shaken well. Finally, 5 μL of PI was added to these cells; the reaction was incubated for 40 min in the dark and analyzed using FACSCalibur (Becton Dickinson).

## Abbreviations

HCPT: Hydroxycamptothecine; VP-16: Etoposide; MTT: 3-(4, 5-dimethylthiazol-2-yl)-2,5diphenyltetrazolium bromide; AO: Acridine orange; EB: Ethidium bromide; TUNEL: Terminal deoxynucleotidyl transferase-UTP nick end labeling assay.

## Competing interests

The authors declare that they have no competing interests related to the work described in this article.

## Authors' contributions

WY, JW performed the experiments, analyzed the data and wrote the paper. L-HJ, DH planned and analyzed the data and B-AS, JW planned the experiments, analyzed the data and wrote the paper. All authors contributed to this study, read and approved the final manuscript.
